# Visual Odometry Using Pixel Processor Arrays for Unmanned Aerial Systems in GPS Denied Environments

**DOI:** 10.3389/frobt.2020.00126

**Published:** 2020-09-29

**Authors:** Alexander McConville, Laurie Bose, Robert Clarke, Walterio Mayol-Cuevas, Jianing Chen, Colin Greatwood, Stephen Carey, Piotr Dudek, Tom Richardson

**Affiliations:** ^1^Flight Lab, Department of Aerospace Engineering, University of Bristol, Bristol, United Kingdom; ^2^Visual Information Laboratory, Department of Computer Science, University of Bristol, Bristol, United Kingdom; ^3^Department of Electrical Engineering and Electronics, The University of Manchester, Manchester, United Kingdom; ^4^Perceptual Robotics, Bristol, United Kingdom

**Keywords:** UAS, navigation, GPS denied, pixel processor array, visual odometry, SIND, Parallel Processing

## Abstract

Environments in which Global Positioning Systems (GPS), or more generally Global Navigation Satellite System (GNSS), signals are denied or degraded pose problems for the guidance, navigation, and control of autonomous systems. This can make operating in hostile GNSS-Impaired environments, such as indoors, or in urban and natural canyons, impossible or extremely difficult. Pixel Processor Array (PPA) cameras—in conjunction with other on-board sensors—can be used to address this problem, aiding in tracking, localization, and control. In this paper we demonstrate the use of a PPA device—the SCAMP vision chip—combining perception and compute capabilities on the same device for aiding in real-time navigation and control of aerial robots. A PPA consists of an array of Processing Elements (PEs), each of which features light capture, processing, and storage capabilities. This allows various image processing tasks to be efficiently performed directly on the sensor itself. Within this paper we demonstrate visual odometry and target identification running concurrently on-board a single PPA vision chip at a combined frequency in the region of 400 Hz. Results from outdoor multirotor test flights are given along with comparisons against baseline GPS results. The SCAMP PPA's High Dynamic Range (HDR) and ability to run multiple algorithms at adaptive rates makes the sensor well suited for addressing outdoor flight of small UAS in GNSS challenging or denied environments. HDR allows operation to continue during the transition from indoor to outdoor environments, and in other situations where there are significant variations in light levels. Additionally, the PPA only needs to output specific information such as the optic flow and target position, rather than having to output entire images. This significantly reduces the bandwidth required for communication between the sensor and on-board flight computer, enabling high frame rate, low power operation.

## 1. Introduction

To achieve successful autonomous operation of UAS, it is necessary for the vehicle to maintain an acceptable estimation of its position. However, weight and power restrictions can place a severe limitation of the on-board sensor equipment which can be used. Sensors commonly found on-board often include a GPS, Inertial Measurement Unit (IMU), and camera. Under most circumstances this is sufficient as the GPS localizes position, while the IMU determines orientation, angular velocities, and linear accelerations. However, there are situations in which the GNSS signals cannot be received, such as underground, indoors, or in hostile GNSS-denied environments. Further there are environments where GNSS signals may be degraded such as in urban or natural canyons. In these situations additional on-board sensors must be used to navigate and maintain knowledge of current position. These can be based on a wide range of technologies such as radar and LIDARs however, one key strategy is the use of cameras for Visual Odometry (VO).

For GNSS-challenging environments, research has been conducted on methods to account for and overcome the degraded signal. Sensor fusion approaches using monocular or stereo-vision cameras alongside the Inertial Navigation System (INS) coupled with the GNSS signal have been tested by Li et al. (2019) and Andert et al. (2013). Other approaches have used multiple vehicles in communication with each other to improve navigation, using those vehicles under normal GNSS conditions to improve the position estimation of any vehicle with poor GNSS signal. This has been approached using a Unmanned Ground Vehicle (UGV) by Sivaneri and Gross (2018), a similar idea was used by Causa et al. (2018) with multiple UAS. Finally Vetrella et al. (2018) used two UAVs in communication, a “father” drone with good GNSS signal, and a “son” which receives an approximate position based on the position of the father and an estimate of distance between the two from a visual approximation. The son uses this data to correct for drift in its own vision based navigation. While not explicitly focusing on GNSS-challenging environments, approach taken in this paper could be used to help improve navigation when facing intermittent or poor GNSS signal.

Focusing on environments where there is no GNSS signal available, using only an Inertial Navigation Systems (INS) can lead to unacceptable drift during extended signal outages as shown by Lasmadi et al. (2017). Visual odometry(VO) can be used to combat this drift, and is attractive as on board cameras are a regular feature of UAS. Specific integrated sensors have also been developed to provide optical flow which can be utilized in for odometry estimation. The PX4FLOW sensor is one such example as shown by Honegger et al. (2013). They have previously been used as part of an integrated navigation system, being combined with INS and magnetometer data in GPS-denied environments for a Micro Air Vehicle (MAV) by Shen et al. (2016) and also as part of a multi-sensor hover control system for multirotors by Ma et al. (2019).

Standard cameras capture a great deal of information in each frame, but struggle with high speed motion due to their relatively low frame rates. Event cameras, which transmit changes of intensity on a pixel by pixel basis (when changes occurs), have latency in the order of microseconds and a High Dynamic Range (HDR) of 140 dB compared to a standard camera at 60 dB (Vidal et al., 2018). This low latency and high dynamic range allow such cameras to function effectively through high speed maneuvers and under poor lighting conditions. They do however struggle when there is limited change in intensity such as small or slow movements. An approach combining event cameras with standard cameras and inertial measurements to produce a more robust result has been demonstrated by Vidal et al. (2018). Due to the limitations of VO with conventional cameras in low light or night time flying, methods involving multi-spectral cameras have also been shown to be an effective approach by Beauvisage et al. (2016).

This paper demonstrates the use of a Pixel Processor Array (PPA) camera for Visual Odometry, working in conjunction with inertial data gathered in flight. The potential of a PPA in comparison with an event camera or a more traditional image sensor is that it can run multiple algorithms directly on sensor device, at high frame-rates, low power (≈ 2*W*), and without the use of additional onboard computation power (Bose et al., 2017). The SCAMP-5 PPA used in this work features an array of 256 × 256 Processing Elements (PE) tightly integrated into the sensor array. A programmable controller provides each PE identical instructions which are carried out simultaneously on all pixels of the image in an SIMD manner. Computer vision algorithms can be directly computed on the pixel array without transferring the image data out of the device, allowing for higher frame rates than conventional systems, potentially making VO through high speed maneuvers possible. Many VO solutions such as that performed by the SCAMP-5 in this paper, are based on the optical flow of the ground beneath the vehicle. As such they require the distance to the surface below to convert visual flow into distance traveled. Therefore, systems such as ultrasonic distance sensors or a laser rangefinder are often used determine height.

In GPS denied environments the appeal of VO is clear for both indoor and outdoor use as argued by Chowdhary et al. (2013). In particular GPS canyons or jamming can pose serious challenges for a platform that relies on only that information. There are a number of ways to approach VO and determine the ego-motion of the system. One popular approach for drones is to rely on monocular vision as discussed in Weiss et al. (2013). Direct VO performs image alignment as the vehicle moves, often working directly on the pixels, allowing distance traveled to be determined. For real time applications GPUs are often used to give the required performance (Shan et al., 2016) however, these bring with them additional weight and power requirements. Feature based VO relies on matching salient features instead of attempting to match an entire image, tracking these features across multiple frames in order to determine sensor motion. Outside of purely visual odometry, approaches using combinations of sensors such as in Visual Inertial Odometry (VIO), where VO is combined with IMU data, have been used to overcome some of the weaknesses associated with both inertial and visual odometry. This was previously used on a vehicle with stereo visual odometry and IMU, producing tracking with an end point distance of 1% of the track length from the ground truth (Kelly et al., 2007). VIO can be used to develop a more robust system as done so by Bloesch et al. (2015) through the use of an Extended Kalman Filter (EKF).

Other approaches to navigation without GNSS include using cameras to localize position using natural landmarks of known position alongside visual odometry as shown for high altitude UAS in Caballero et al. (2014) and on small UAS in GPS denied environment in Wang et al. (2012). Simultaneous Localization and Mapping (SLAM), while more computationally expensive, is another potential approach to overcoming this problem. A single camera alongside an IMU has previously been used to determine real time 6 degrees of freedom localization, navigation, and obstacle avoidance in a forest environment by Langelaan and Rock (2005). Other approaches to SLAM use a scanning LiDAR to build a 2D or 3D point cloud or occupancy map of the environment, has been used alongside IMU data to produce a more integrated navigation system by Li et al. (2014).

Beyond the continuous state estimation of world and platform as performed in SLAM, other works have started to combine visual state estimation with recognition. One well integrated competence is re-localization, which is a form of object recognition when treating space as a 3D object to be detected as in Martinez-Carranza et al. (2013) and Martínez-Carranza et al. (2016). In Bartholomew et al. (2012), object materials are visually recognized to predict landing behavior. Recent approaches also extend visual localization combining VO or SLAM with neural networks. Neural networks have been used to overcome a wide variety of challenges involved in visual localization, from coping with dust and fog occlusion (Kubelka et al., 2019), changes in environment due to lighting and weather (von Stumberg et al., 2020), and improving depth estimation (Feng and Gu, 2019).

This paper demonstrates the use of visual odometry and target identification running on-board the SCAMP-5 PPA during flight. Multiple runs are shown, with direct comparison of the VO estimation against the baseline GPS results. Whilst computing VO, the PPA also runs a target identification algorithm in parallel. This target identification allows the position estimate to be updated when located, and demonstrates the use of multiple algorithms running on-board a single PPA chip. In future work further additional algorithms could be included, such as perspective correction (Greatwood et al., 2018), all without the need for additional computation hardware. Results are also given for the commercially available PX4FLOW optical flow sensor, showing SCAMP-5 can provide comparable VO results while offering the potential for significant additional capabilities.

## 2. Methods

### 2.1. Hardware

#### 2.1.1. SCAMP-5 Pixel Processor Array

**Figure 1:**
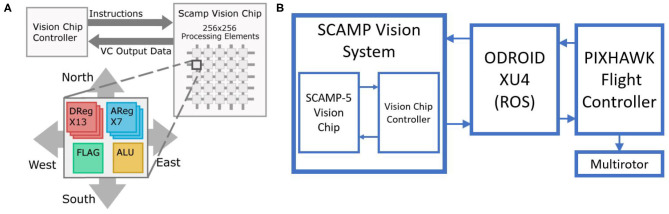
**(A)** Architecture of the SCAMP-5 vision system, with each pixel having storage and processing capabilities (Bose et al., 2017), **(B)** Block diagram of the system (Greatwood et al., 2018).

Figure 1 illustrates the architecture of the PPA and how it is integrated into the multirotor. Each Processing Element (PE) in the 256 × 256 array is able to capture, store, and process visual data. These effectively act as a simple “microprocessors” for every pixel in the array. The PEs have a photo-sensor, local analog and digital memory, and are able to perform logic and arithmetic operations with data transfer between PEs also possible (Bose et al., 2017). By only transmitting data such as the sensor's ego-motion out of the array, while running computation internally, required bandwidth and power are significantly reduced. This allows visual tasks to be carried out at high frame rates, greater than 1,000 Hz (Bose et al., 2017). The flight test speeds however did not require such a frame rate, and so frequency was reduced to 200 Hz for our experiments to reduce the size of recorded data logs. However, the capability to function at such high frame rates allows SCAMP to be used at significantly greater flight speeds and through high speed maneuvers experiencing minimal image motion blur. The complete SCAMP-5 system requires approximately 2 watts of power (Bose et al., 2017), making the PPA an excellent option for computer vision algorithms on smaller low power vehicles. The unit is currently housed in a 3D printed case weighing 100 g and measuring 82 mm × 77 mm 23 mm without the lens.

#### 2.1.2. PX4FLOW Sensor

Optical flow cameras work by tracking the motion of objects, edges, or surfaces within the frame. This particular sensor was chosen for comparison with the SCAMP-5 sensor, having a native resolution of 752 × 480 pixel and using 4× binned and cropped area at 400 Hz to track motion. Integrated on-board is an ultrasonic distance sensor however the upper limit to the range of this sensor is in the region of 3.5 m, limiting its value for our application where the trials were carried out at a heights of 5 or more meters Above Ground Level (AGL). Data gathered by laser rangefinder was therefore used in place of this sonar when comparing the results.

#### 2.1.3. Multirotor Platform

**Figure 2:**
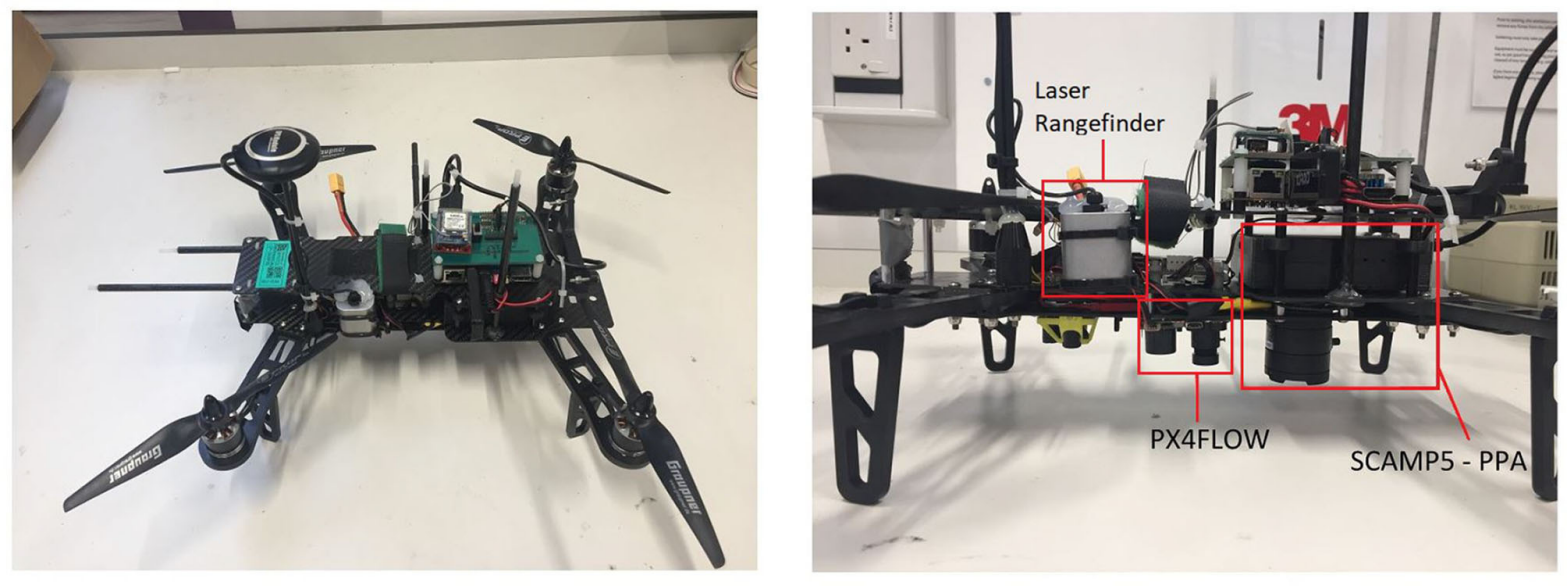
Multirotor platform used as the SCAMP-5 test-bed, with ODROID XU4 and PX4FLOW sensors.

A custom multirotor platform shown in Figure 2 was developed for testing the VO system, allowing the SCAMP-5 sensor to be attached facing forwards or vertically downwards as desired. The multirotor weighs 1 kg with SCAMP-5 installed and measures 400 mm diagonally between rotors. For the following work the SCAMP-5 was deployed in the downward facing configuration. On-board the multirotor, an ODROID XU4 single board Linux computer which enables both SCAMP-5 and the Pixhawk flight controller to be integrated. It should be noted that the primary role of this computer is that of communication and that the VO and target identification is being carried out on board the SCAMP-5 PPA chip itself. Additionally a laser rangefinder has been added to the frame to allow the height above ground to be determined.

### 2.2. SCAMP-5 Algorithms

This section briefly describes the visual odometry algorithm executed upon the SCAMP-5 sensor, based upon the previous work of Bose et al. (2017), along with the algorithm used to identify and extract a specific target pattern as previously used in Greatwood et al. (2017).

#### 2.2.1. Image Alignment Based Visual Odometry

The visual odometry used in this work was conceived to investigate what tasks are possible to conduct entirely on-sensor using a PPA without relying on external hardware for processing. As such it does not provide a full 6DOF odometry solution, instead providing 4 readings related to how the observed image content rotates, scales and translates from one frame to the next. The rotation measurement is related to sensor roll, and the scaling measurement to forward and backward translation along the sensor's axis. The measurements for how the image is translating between frames can be associated with both changes in sensor orientation or sensor translation parallel to the image plane. These last two measurement thus require either constraints on sensor motion, or additional information to solve this ambiguity (In this work specifically, IMU data is used to address this issue).

The SCAMP-5 sensor is mounted facing downwards under the vehicle observing the ground below. In an ideal situation where the sensor's orientation remains locked facing normal to the ground, the translation measurements from the odometry correspond to X and Y translation parallel to the ground plane, rotational component to vehicle yaw (sensor roll), and scaling to sensor height. However, in practice the vehicle will change orientation during flight breaking this assumption of fixed sensor orientation. For example changes in roll and pitch of the vehicle will result in the sensor's view sweeping across the ground creating false measurements of translational motion from the visual odometry. Because of this the vehicle's IMU sensor data must be combined with the visual odometry output during flight to correct for such ambiguities. In practice this is done by converting the IMU rotation that occurred in a time step into the distance covered using knowledge of height *h* using the following equation where Δθ is the change in pitch of the vehicle and Δϕ is the roll, while ω_*x*_ and ω_*y*_ are the distance perceived in the respective body axes caused by vehicle rotation. A laser range finder on the vehicle is also used to convert the odometry's translation estimate from pixel measurements, to actual distance traveled.

(1) $$\begin{array}{l} \left[\begin{array}{l} \omega_{x}\\ \omega_{y} \end{array}\right]=\left[\begin{array}{l} tan\left(\Delta \theta \right)\\ tan\left(\Delta \phi \right) \end{array}\right]h \end{array}$$

**Figure 3:**
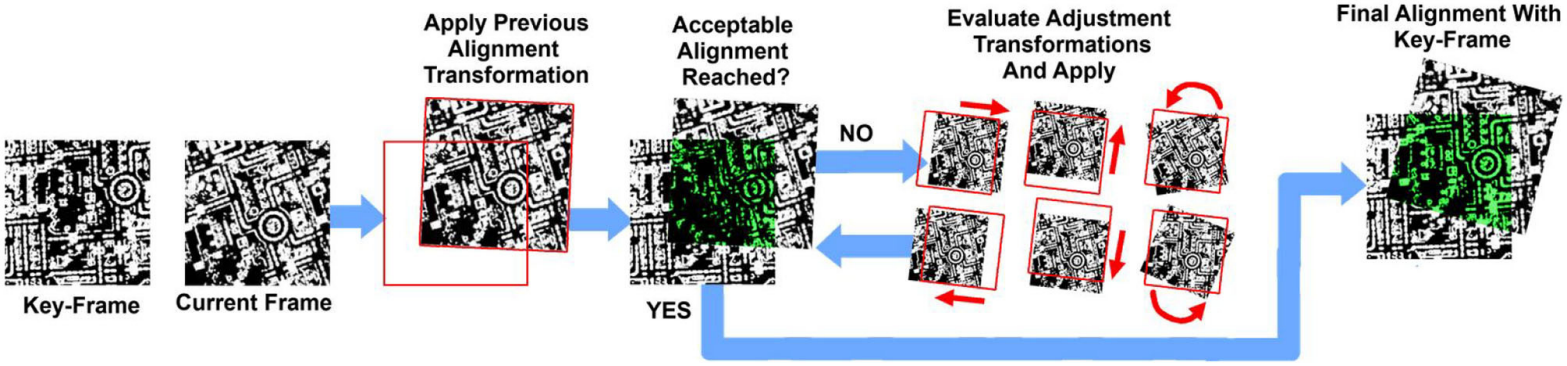
Diagram showing the steps of the image alignment process performed upon SCAMP-5. First the transformation which achieved alignment for the previous frame is applied to the current frame. Many small adjustment transformations are then evaluated, those which improve alignment are then applied. This process is repeated until alignment strength is deemed acceptable.

This PPA odometry utilizes a image alignment based approach, whereby each captured image of the ground below is aligned with a previously captured “key-frame” image. The transformation determined to achieve this alignment consists of rotation, scaling, and translation components, each associated with a different form of sensor motion as described previously. The process of determining this alignment transformation, is conducted entirely upon the SCAMP-5's PPA array using an iterative approach similar to gradient descent, described in greater detail in Bose et al. (2017). In each alignment iteration four pairs of small adjustment transformations are then tested, each pair being in opposite “directions.” These transformations being left and right image translations, up and down image translations, clock-wise and anti clockwise image rotations, and up-scaling and down-scaling transformation. Each transformation in a pair is evaluated to determine which of the two possible transformations would improve image alignment, using the strength metric described below. As multiple small transformations are applied the resulting image transformation converges toward one which aligns the two images. Note that in practice the alignment transformation determined for the previous frame is used as the initial transformation to start from in the current frame, this allows image alignment to be typically achieved in a single iteration. The steps of this alignment process are illustrated in Figure 3. Combined with the fact the entire process is performed upon the SCAMP-5 PPA, this visual odometry approach can achieve frame rates in excess of 1,000 Hz given sufficient lighting conditions.

**Figure 4:**
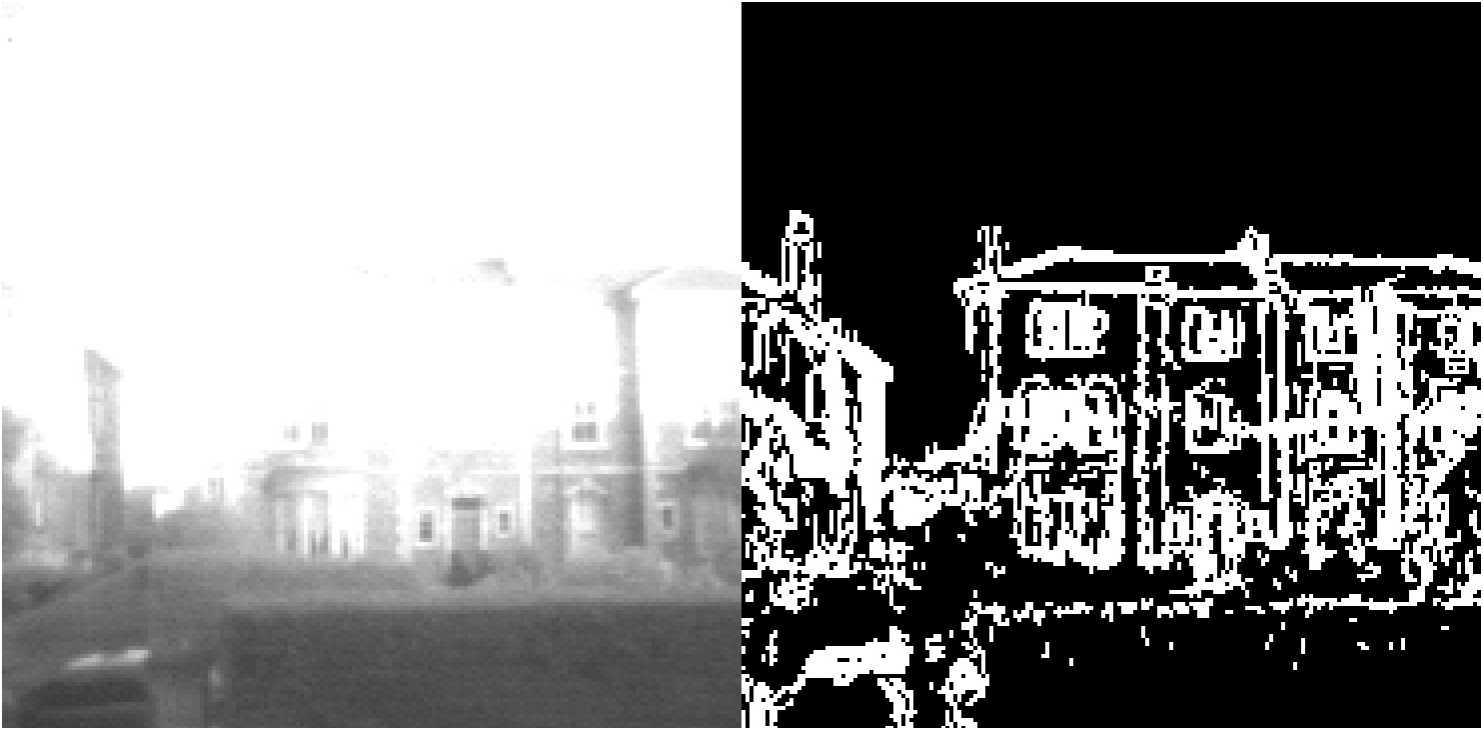
**(Left)** Overexposed image captured by SCAMP-5. **(Right)** A binary image resulting from HDR edge extraction being performed during the capture of the left image.

Rather than raw images, this process makes use of HDR edge images as shown in Figure 4, which are both more invariant to changes in lighting conditions and result in fewer incorrect local minima for the alignment process to converge toward. Evaluating the “strength” of alignment between two HDR edge images involves some measure of edges shared between both images, present in the same locations. The approach we use involves first performing an AND operation between the two edge images on SCAMP-5, eliminating all white pixels that are not shared between both images. These remaining shared edge pixels can then be counted and divided by the total number of white edge pixels from the key-frame image giving a measure of the alignment strength.

In practice the visual odometry is triggered to start once the vehicle has taken off and put into a hover state, capturing an initial key-frame of the ground below. As the vehicle moves through the environment each new frame is aligned to the current key-frame and the strength of alignment evaluated. Once alignment strength has fallen below a given threshold a new key-frame is acquired, replacing the existing one and allowing the odometry estimation to continue.

#### 2.2.2. SCAMP-5 Target Extraction

**Figure 5:**
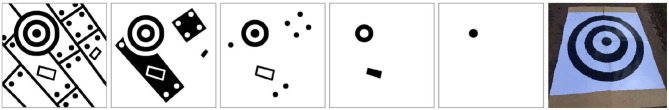
Target extraction algorithm stages, showing the result of performing 4 “flood and invert” operations starting from an initial image on the left, until only the center region of the target remains in the fifth frame, and an image of the artificial target in position during testing.

The target pattern and extraction algorithm used in this work is designed to exploit one of the native features of the SCAMP5-PPA: fast parallel pixel flooding, performing a flood fill operation on a stored binary image. Beginning from a set of selected pixels, this flooding operation spreads across any connected region of black pixels, setting their content to white. The target extraction algorithm performs flooding starting from all pixels along the borders of the image. This effectively eliminates all regions that are not enclosed by a boundary of white pixels. By inverting the image and repeating this process, all regions that were not enclosed within two distinct boundaries in the original image will be filled in. By repeating this sequence of flood and invert operations *N* times, all regions not contained within more than *N* boundaries are filled in and effectively eliminated from the image. Thus, by using a target pattern consisting of a large number of boundaries, repeated flood and invert operations can reliably be used to eliminate all content from the image except some inner region of the target, even in visually cluttered environments. This concept is illustrated in Figure 5. The bounding box of this remaining region can then be extracted from the image, providing the target's location.

Note this same approach could be modified to track multiple targets, each consisting of a different number of boundaries. This would involve first performing sufficient flood and invert operations such that any remaining image content belongs to a target. The bounding boxes of each remaining target can then be extracted to give their target's location. Performing further flood and invert operations will then eliminate the remaining targets from the image, starting with that of the fewest boundary layers. Each target present in the image can then be identified by the number of flood and inverts operations performed to eliminate it.

### 2.3. Flight Tests

**Figure 6:**
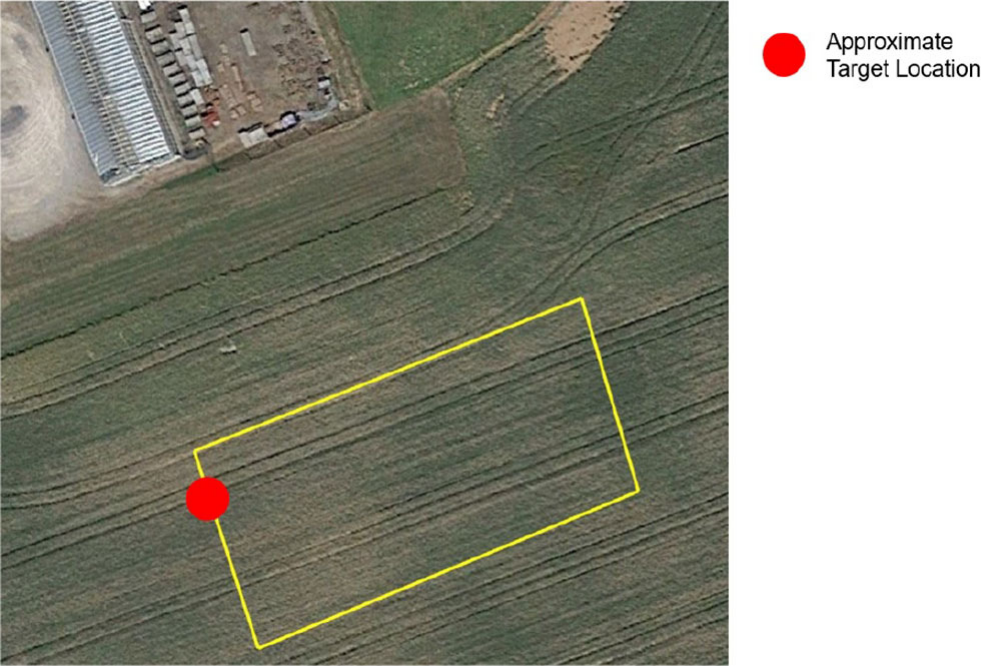
GPS way point path shown overlaid at the test location with target location identified—approximately 50 m × 25 m.

Outdoor flight paths were tested to evaluate the SCAMP-5 visual odometry for use on UAS. Initial flight tests involved flying automated rectangular paths, yawing 90 degrees at each corner at 5 m AGL and at 3 m·s^−1^. The flights took place over a field shown in Figure 6, which itself could pose a difficulty for the SCAMP-5's imaging capabilities as the ground may appear to be featureless or too feature dense depending on altitude. The SCAMP-5 VO data was post processed to include the yaw, pitch and roll captured by the IMU to account for the rotation of the vehicle, with the odometry running at 200 Hz. Additionally the target extraction algorithm was run alongside the visual odometry, with the parallel processing capabilities of the SCAMP-5 allowing both algorithms to run at 200 Hz. This was used to correct the SCAMP-5 odometry, by having the target positioned at a known location. The target in question was an artificial pattern not a natural feature as shown in Figure 5.

**Figure 7:**
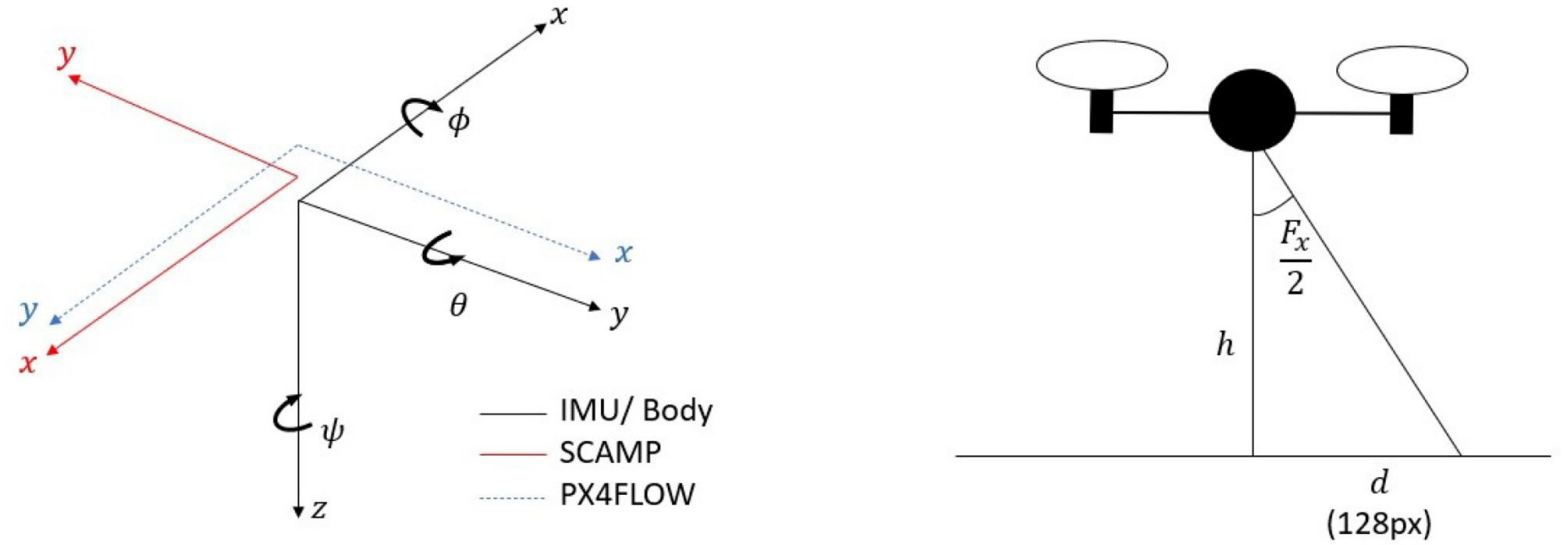
Diagram showing the sensor orientations and a diagram highlighting the system approximations used to determine the distance value of each pixel.

All data was converted into an North-East-Down (NED) inertial frame for comparison between the PX4FLOW and GPS. Figure 7 shows the orientations of the sensors relative to the body frame.

#### 2.3.1. SCAMP-5 Vision System

The odometry data computed by the SCAMP-5 system is in the sensor's reference frame and given as pixels moved since the last reading. These pixels were converted to distances by using the Field of View (FOV) of the lens (≈ 50°), a scaling function and, the sensor size (256 × 256 pixels) to determine the distance each pixel represents. *F*_*x*_ and *F*_*y*_ are the field of view of the lens in the *x* and *y* directions of the SCAMP frame respectively,*f*(*h*)_*x*_ and *f*(*h*)_*y*_ represent the non-linear scaling function that varies with height, while *d*_*xO*_ and *d*_*yO*_ are distance values in the *x* and *y* directions traveled in the SCAMP frame since the previous reading, and *x*_*O*_ and *y*_*O*_ are the pixel distances moved since the previous reading. The attitude of the body is assumed to be small throughout the flight and is therefore ignored allowing us to use equation (2) based on the second image shown in Figure 7 and simple trigonometry we are able to determine an approximation for the distance value of a pixel for the SCAMP system at a given time. This does not take into account perspective effects, and other sources of error which we correct for using the scaling function.

(2) $$\begin{array}{l} \left[\begin{array}{l} d_{xO}\\ d_{yO} \end{array}\right]=\frac{h}{128}\left[\begin{array}{l} tan\left(0.5F_{x}\right)\\ tan\left(0.5F_{y}\right) \end{array}\right]\left[\begin{array}{l} x_{O}\\ y_{O} \end{array}\right]\left[\begin{array}{l} f{\left(h\right)}_{x}\\ f{\left(h\right)}_{y} \end{array}\right] \end{array}$$

As previously described by Equation (1) ω_*x*_ and ω_*y*_ are the components of motion produced by the rotation of the body.

(3) $$\begin{array}{l} \left[\begin{array}{l} d_{xVIO}\\ d_{yVIO} \end{array}\right]=\left[\begin{array}{l} d_{xO}\\ d_{yO} \end{array}\right]-\left[\begin{array}{l} \omega_{y}\\ \omega_{x} \end{array}\right] \end{array}$$

To convert the data into the inertial frame the following transformation is applied, consisting of the standard rotation matrix about the *z* axis as seen in McRuer et al. (1973). The transformation has been adjusted to account for the difference in reference frame between the SCAMP and the IMU.

(4) $$\begin{array}{l} \left[\begin{array}{l} d_{xVIO}^{I}\\ d_{yVIO}^{I} \end{array}\right]=\left[\begin{array}{c} -cos\left(\psi \right),sin\left(\psi \right)\\ -sin\left(\psi \right),-cos\left(\psi \right) \end{array}\right]\left[\begin{array}{l} d_{xVIO}\\ d_{yVIO} \end{array}\right] \end{array}$$

Finally to determine the position throughout the flight $x_{VIO}^{I}$ and $y_{VIO}^{I}$ the steps $d_{xVIO}^{I}$ and $d_{yVIO}^{I}$ are summed.

(5) $$\begin{array}{l} \left[\begin{array}{l} x_{VIO}^{I}\\ y_{VIO}^{I} \end{array}\right]=\Sigma_{t}\left[\begin{array}{l} d_{xVIO}^{I}\\ d_{yVIO}^{I} \end{array}\right] \end{array}$$

The resulting path following the IMU correction and transformation through the IMU yaw could be considered a type of Visual Inertial Odometry (VIO) and is referred to as such from here on. A similar transformation is carried out on the PX4FLOW data captured during the flight however IMU correction is not performed as the PX4FLOW has a gyroscope built in, and a different scaling factor was determined.

### 2.4. Visual Odometry and Target Extraction

Tests were performed running both visual odometry and target extraction upon SCAMP-5 to determine the effect of running two algorithms simultaneously. The same flight path from the initial odometry tests were used, however a target as shown in the final frame of Figure 5 is placed by one corner of the path as shown in Figure 6. The purpose of this test is to both demonstrate the capability of the work done by Greatwood et al. (2017), and demonstrate multiple algorithms running on-board the SCAMP-5. We correct the position of the odometry when the target is detected, using for demonstration purposes an assumed target position and zero error in the distance estimation upon detection.

We note that while the current SCAMP resolution and lens used results in an approximate 0.2° per pixel, which may appear low, it is an order of magnitude higher than other successful flying platforms such as honey bees that also demonstrate navigation and recognition (Avargues-Weber et al., 2010).

## 3. Results

### 3.1. Flight Tests

Whilst an approximation for VO contributions due to rotations of the vehicle can be directly calculated, it is more difficult to calculate from fundamentals the non-linear relationships that exists between the VO measured by the SCAMP sensor and the true distance covered by the aircraft. Factors which contribute to this scaling include lens effects, both across the sensor plane and as a function of height; any misalignment with the body axes of the aircraft and any inherent differences of the sensor related to the optic flow direction and perspective effects. Because of this, an experimental approach was taken in order to find the nonlinear scaling function to account for sources of error in the SCAMP-5 system and lens combination. This non-linear scaling function was determined by manually scaling data that was not used in the analysis to match the GPS track at different altitudes, the scaling factors at these altitudes where then used to develop a scaling function that varied with height. A scaling factor was also applied to the PX4FLOW data which was determined in the same way as each individual scaling factor for the SCAMP, this was required as the operating altitude of 5 m AGL was outside of the PX4FLOW sensors range capability. However, a height scaling function was not developed for the PX4FLOW as the comparison was completed for a run at an approximately consistent 5 m altitude. Note that accurate state knowledge is required to determine this factor initially, but the same factors were applied to all data analyzed. Further work could be done to define a function for this sensor and lens combination in future however, that is outside the scope of this work.

**Figure 8:**
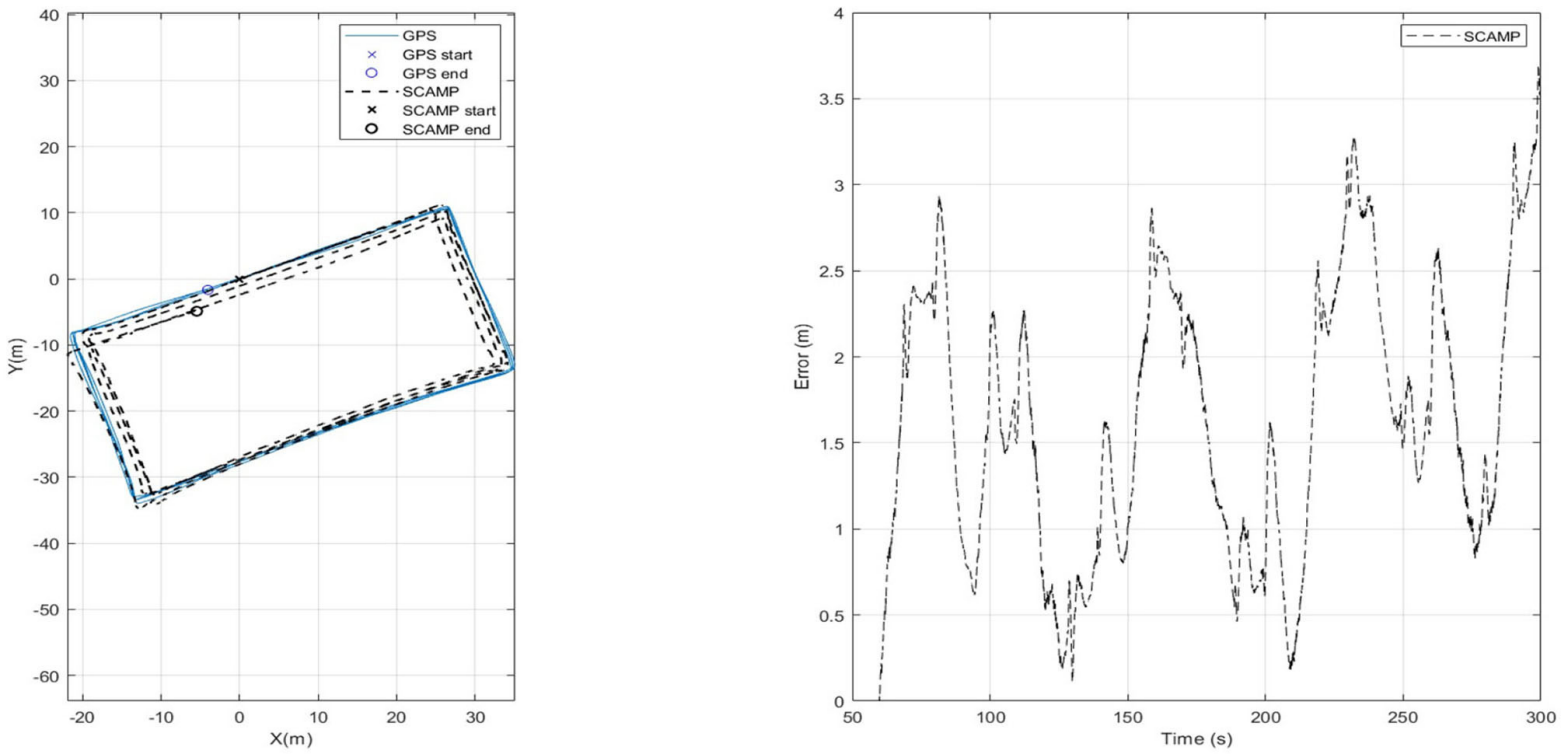
Scaled flight path completing circuit four times and absolute error plot.

A rectangular path of 50 m × 25 m was repeated four times as shown in Figure 8. Each loop was assessed independently and then combined to demonstrate the performance of the system over a longer time period as shown in Figure 8, the right hand side of which shows the error to increase slowly, remaining below 4 m over a total distance of 620 m. All flight tests over this rectangular path involved a 90 degree yaw at each corner, and were flown at 3 m·s^−1^. The results of each lap and the combined track are summarized in Table 1.

**Table 1:** Sensor performance on circuits after scaling is applied.

**Circuit no**.	**Track length diff (%)**	**Mean Abs error (m)**	**End point dist (m)/(% track length)**
1	−5.12	1.63	0.61/0.39
2	−3.01	1.30	1.07/0.69
3	−3.40	1.38	1.43/0.92
4	−4.15	2.81	3.76/2.47
1–4 Combined	−3.90	1.63	3.53/0.57

### 3.2. Visual Odometry and Target Extraction

**Figure 9:**
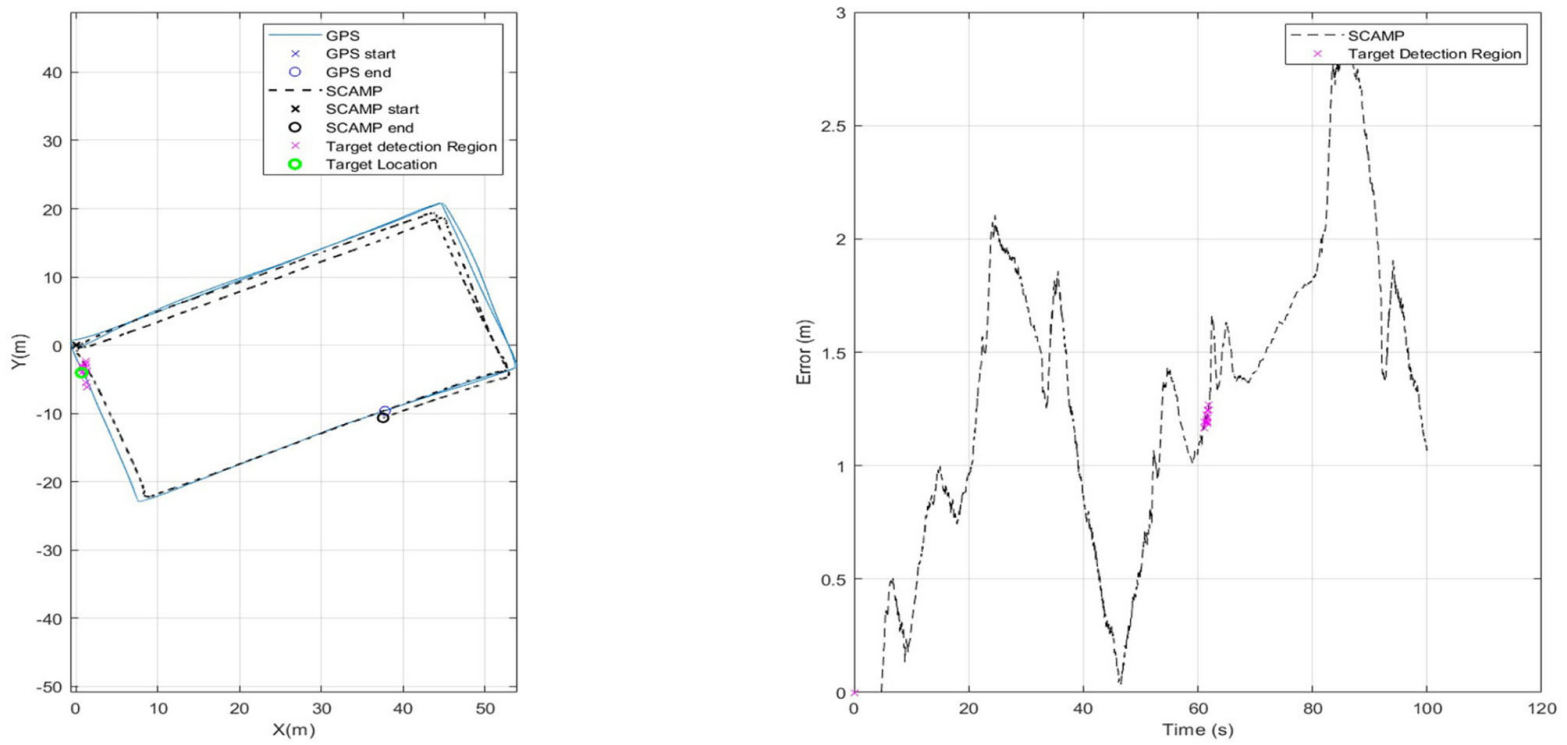
Plot showing testing of target extraction algorithm in flight. Target location shown with a green circle, and magenta indicating when SCAMP identified the target.

For the next set of results shown in Figure 9, the SCAMP-5 was given a second algorithm, target extraction, to run concurrently with the VO each running at 200 Hz. It should be noted that the term concurrently in this context means sequentially for each algorithm is applied to each individual frame. The multirotor followed the same predefined path used to test the visual odometry in isolation however, a target shown in the final frame of Figure 5 approximately 1m in diameter was placed on the ground by one corner of the flight path. This target extraction was previously integrated into the control loop of a multirotor to track and follow a moving target (Greatwood et al., 2017). Figure 9 shows a flight path with the region where the target is identified in parallel with the VIO track being estimated. When the SCAMP detects the target its pixel coordinates in image frame are returned to the on-board computer. For this run, the VIO error is increasing slowly, but it is clear when the SCAMP can identify the target in view. For the next set of results detection of the target is used to correct the current drift error of the VIO estimate of position.

**Figure 10:**
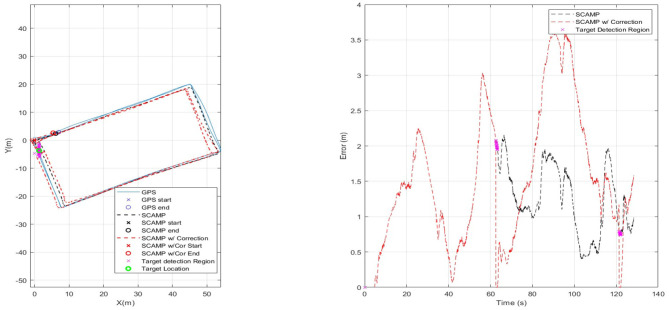
Using a known target location to remove current SCAMP VO error.

If the position of the target is known, then the error that has built up in the VIO estimate can be reduced to the uncertainty in the position of the target whenever it is detected by the SCAMP. For the results shown in Figure 10 perfect knowledge of the target position was assumed and used to correct the odometry position to that of the GPS whilst the target was visible—as can be see in the right hand plot in Figure 10. We envision possible future operations, in which a sensor array including PPA based vision chips can be used to generate the estimated position during flight, which is updated as known markers, locations, or objects are identified. This may be through simple target detection as done here or through more complex approach such as neural network based identifiers.

**Figure 11:**
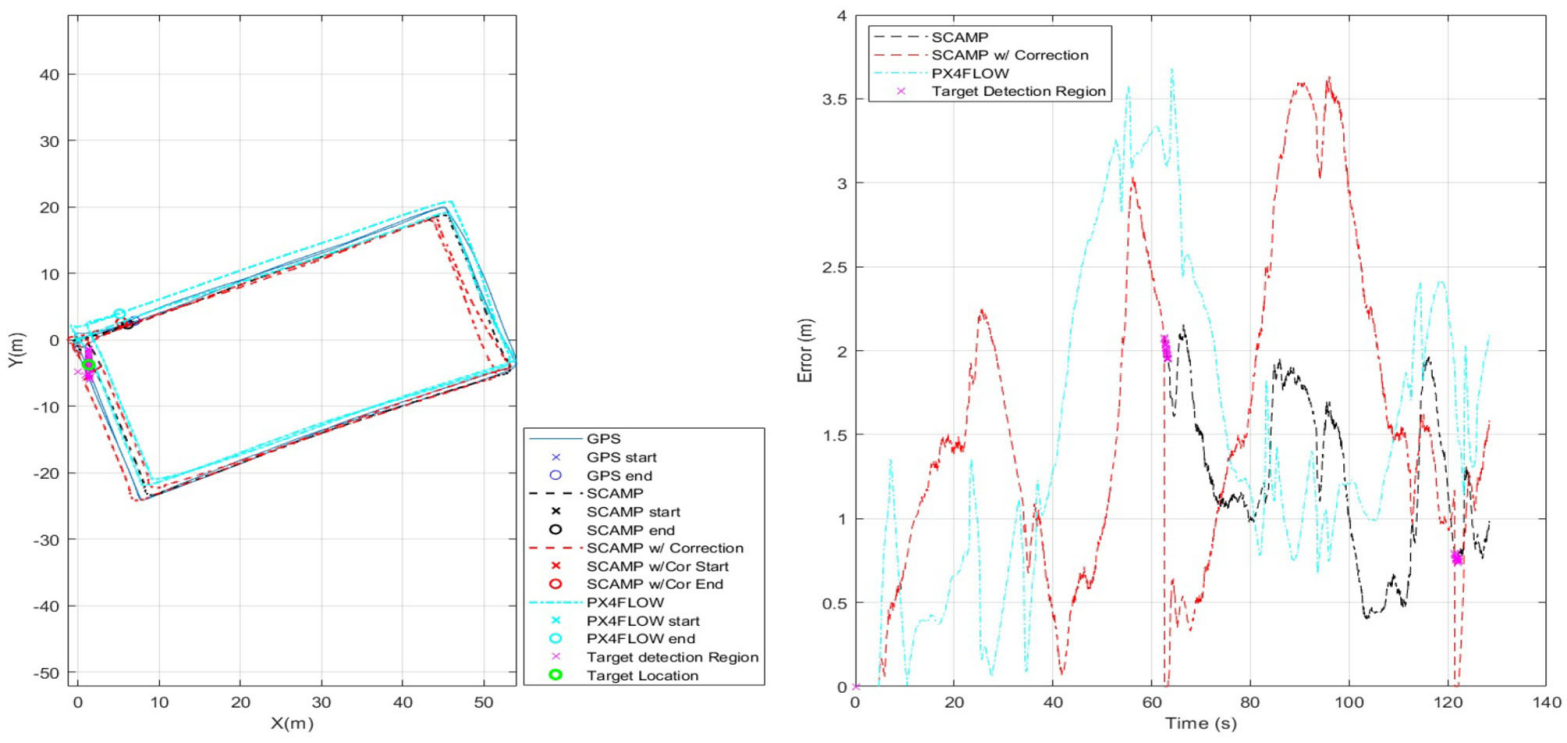
Comparison of results for SCAMP-5, SCAMP-5 with Target Correction and, PX4FLOW against GPS.

Figure 11 has three sets of data for comparison. It has the initial SCAMP-5 VIO estimate in black—which is comparable with data captured using the PX4FLOW sensor on the same flight. Both sensors have similar levels of performance, however the SCAMP-5 PPA also performs target detection, as shown in the red where target detection is used to remove the drift in the estimate twice as the vehicle passed over the target. These results are summarized in Table 2. In this case the mean error is slightly increased by including the target correction. Though the main purpose of this was to show the SCAMP system running multiple algorithms concurrently, the increase in error is likely caused by a combination of the track being cyclical and direction of the drift in the data. If this test was carried out over a long distance track with multiple targets this target correction may prove be of more significant use. As shown in the table, the performance of all three approaches is very similar, each maintaining a mean absolute error of between 1 and 2 m.

**Table 2:** Comparison of results between SCAMP-5, SCAMP-5 w/ Target correction and, PX4FLOW.

**System**	**Track length diff (%)**	**Mean error (m)**	**End point dist (m)/(% track length)**
SCAMP-5	−3.79	1.27	0.98/0.31
PX4FLOW	−1.76	1.54	2.10/0.67
SCAMP-5 w/ Target Cor	−2.58	1.57	1.59/0.51

**Figure 12:**
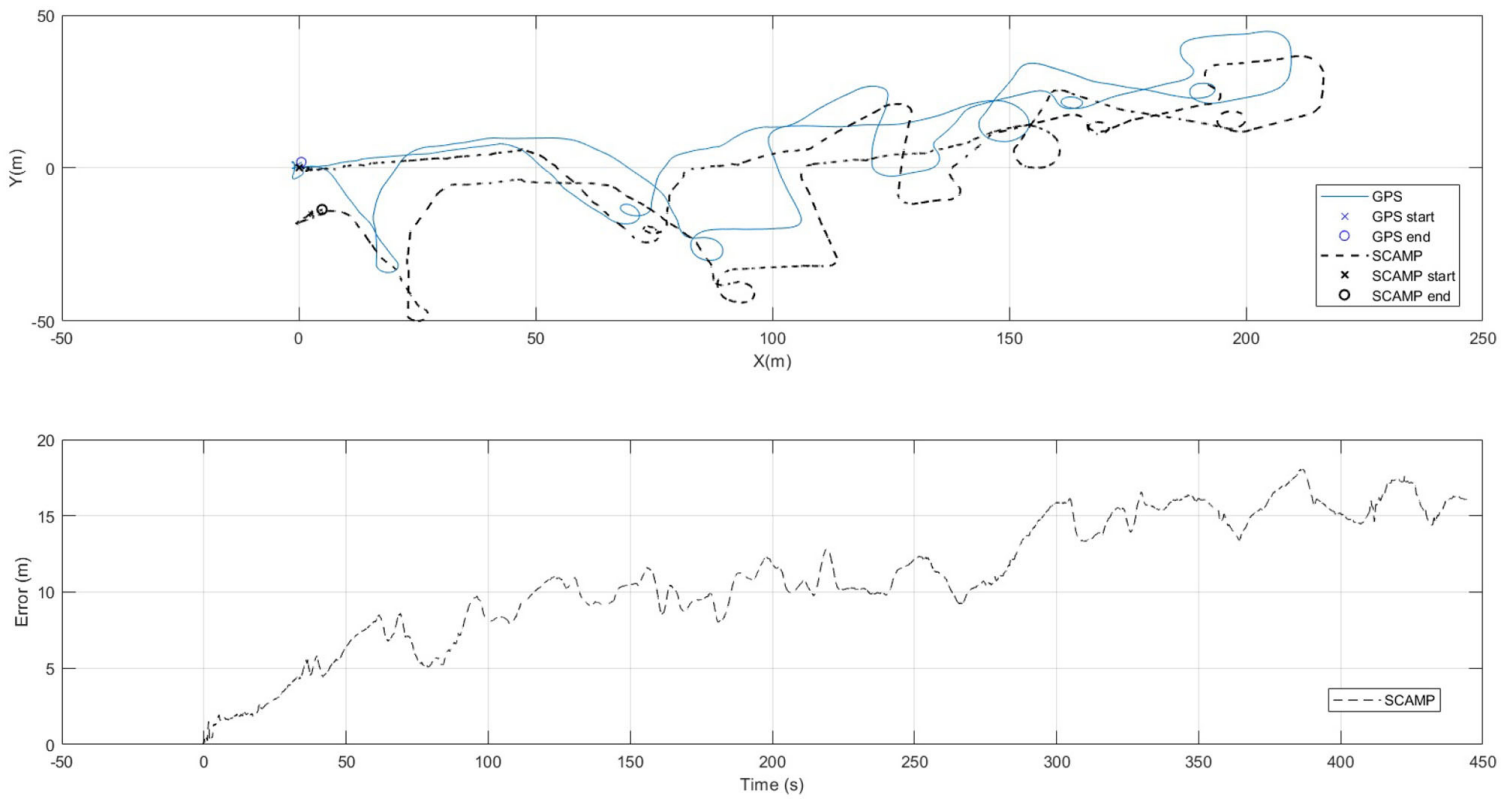
Diagram showing the SCAMP-5 systems performance against GPS at following a complex flight path of approximately 790 m in length.

For Figure 12 a more complex path was flown. The VIO estimate of the position is show in conjunction with the GPS baseline and the absolute error can be seen steadily increases but remains below 20 m over the entire track length of 0.79 km, with and end point distance of just over 15 m.

## 4. Conclusions and Future Work

In this paper we have demonstrated that the SCAMP-5 PPA sensor can be used for position estimation in outdoor flight, potentially enabling navigation and recovery in GNSS-denied environments. The results collected demonstrate performance similar to that of a commercially available PX4FLOW VO sensor, yet offering significant advantages in terms of running additional algorithms at reduced power and weight requirements. On a long, complex track the SCAMP-5 VIO estimate of position was shown to be approximately 2.5% percent of the overall track length of approximately 0.8 km, which could be of use in many scenarios involving the use of UAS in challenging environments.

The SCAMP enables us to complete two tasks using the same sensor. The overall VIO performance did not suffer during the multi-algorithm test due to the high frame rates that were achieved. The use of a second localization algorithm has also been shown to offer the opportunity to reduce or remove positional errors when known targets can be identified. It should be noted though that additional experimental and modeling work is required in order to create a robust nonlinear scaling function for the VIO that is insensitive to significant changes in vehicle orientation, and alignment errors.

By making use of the SCAMP-5's multi-functional capability we can further improve the vehicles navigation capabilities and offer a single low power (≈ 2W) sensor that can provide a wide range of functionality. This has been demonstrated by works on HDR imaging (Martel et al., 2016), feature extraction (Chen et al., 2017), neural network classifiers (Bose et al., 2019), and other on-sensor algorithms which can potentially be used to enhance future VO functionality, creating extremely capable, low weight, low power multi functional sensors. As a proof of concept, this paper demonstrates the potential for use of PPAs in GNSS denied or challenging environments. Future work will focus on reducing the overall integration size and weight, refining the VO algorithm, flight testing using the VO and feature localization as the primary means of navigation; and flight at much higher altitudes and over longer path lengths.

## Data Availability Statement

Due to the nature of the PPA, the data used for evaluation in this work was never recorded. All datasets presented in this study are included in the article.

## Author Contributions

AM completed majority of writing, data analysis. LB operated SCAMP system during flight tests and simulation and also developed and described the algorithms used. RC aided in flight testing and extraction of data. TR, WM-C, SC, and PD gave support and guidance on experimentation, writing, and analysis. CG designed and built the custom vehicle used to house the SCAMP camera. JC aided in the algorithm development. All authors contributed to the article and approved the submitted version.

## Conflict of Interest

CG while working on the project was not employed by Perceptual Robotics though by the completion of the project had begun employment there. The remaining authors declare that the research was conducted in the absence of any commercial or financial relationships that could be construed as a potential conflict of interest.

## Abbreviations

GNSS: Global navigation satellite system
PPA: pixel processor array
PE: processing element
INS: inertial navigation system
IMU: inertial measurement unit
VO: visual odometry
VIO: visual inertial odometry
CV: computer vision
UAS: unmanned aerial system
HDR: high dynamic range
AGL: above ground level
MAV: micro air vehicle
EKF: extended Kalman filter.
